# An exploration of workarounds and their perceived impact on antibiotic stewardship in the adult medical wards of a referral hospital in Malawi: a qualitative study

**DOI:** 10.1186/s12913-019-3900-0

**Published:** 2019-01-23

**Authors:** Chimwemwe Tusekile Mula, Nicola Human, Lyn Middleton

**Affiliations:** 1 Department of Clinical Nursing, University of Malawi, Kamuzu College of Nursing, Blantyre, Malawi; 2 School of Nursing and Public Health, College of Health Sciences, University of KwaZulu-Natal, Durban, South Africa; 3 School of Health Sciences, Department of Pharmacy, University of KwaZulu-Natal, Durban, South Africa

**Keywords:** Standard, Patient safety, Medication administration, Antibiotics, Antibiotic stewardship, Workaround, Improvisation, Quality improvement, Problem-solving, Shortcut

## Abstract

**Background:**

Antibiotic stewardship, the proper management of antibiotics to ensure optimal patient outcomes, is based on quality improvement. Evidence-based guidelines and protocols have been developed to improve this process of care. Safe and timely patient care also requires optimal coordination of staff, resources, equipment, schedules and tasks. However, healthcare workers encounter barriers when implementing these standards and engage in workarounds to overcome these barriers. Workarounds bypass or temporarily ‘fix’ perceived workflow hindrances to achieve a goal more readily. This study examines workaround behaviours that nurses and doctors employ to address the challenges encountered during their antibiotic stewardship efforts and their impact, at a tertiary hospital in Malawi.

**Methods:**

This was a qualitative descriptive case study design and is part of a large mixed methods study aimed at understanding nurses’ role in antibiotic stewardship and identifying barriers that informed the development of nurse-focused interventions. For this study, we conducted interviews with staff and observations of nurses antibiotic stewardship practices on two adult medical wards. We convened three focus group discussions with doctors, pharmacists and laboratory technologists (*n* = 20), focusing on their attitudes and experiences with nurses’ roles in antibiotic stewardship. We also observed nurses’ antibiotic stewardship practices and interactions duringfour events: shift change handovers (*n* = 10); antibiotic preparation (*n* = 13); antibiotic administration (*n* = 49 cases); and ward rounds (*n* = 7). After that, the researcher conducted follow up interviews with purposively selected observed nurses (*n* = 13).

**Results:**

Using inductive and deductive approaches to thematic analysis, we found that nurses established their ways of overcoming challenges to achieve the intended task goals with workarounds. We also found that nurses’ practices influenced doctors’ workarounds. We identified six themes related to workarounds and grouped them into two categories: “Taking shortcuts by altering a procedure” and “Using unauthorized processes”. These behaviors may have both positive and negative impacts on patient care and the health care system.

**Conclusion:**

The study provided insight into how nurses and doctors work around workflow blocks encountered during patient antibiotic management at a tertiary hospital in Malawi. We identified two categories of workaround namely taking shortcuts by altering a procedure and using unauthorized processes. Addressing the blocks in the system by providing adequate resources, training, improving multidisciplinary teamwork and supportive supervision can minimize workarounds.

**Electronic supplementary material:**

The online version of this article (10.1186/s12913-019-3900-0) contains supplementary material, which is available to authorized users.

## Background

Providing safe and timely patient care requires optimal coordination of staff, resources, equipment, schedules and tasks [1]. In response to this demand, health care organisations have implemented standards for different care processes to improve patient safety [2]. One of these processes is medication administration. The administration of antibiotic medications is particularly important, as antibiotic resistance threatens their future utility [3]. Antibiotic stewardship programs which provide standards for antibiotic dose optimisation, have been developed to address this threat [4].These standards have been internationally endorsed [4–6] and appear favourable on paper; however, implementation of favourable on-paper policies in the context of complex real-life contexts is difficult [7, 8]. The context of low and middle-income countries may further challenge their implementation because of limited healthcare resources and competency challenges [9, 10].Nurses find it challenging to implement medication process standards for antibiotic stewardship consistently. Challenges with: drug delivery, increases in the complexity of care and the number of prescribed medications per patient, and the number of patients assigned to each nurse contribute to errors in the process. [11]. Additionally, clinicians who prescribe the medications experience a similar challenge with microbiology laboratory testing for culture and sensitivity and may prescribe an antibiotic before or without a clear test result. As such, health care workers encountering barriers engage in workarounds to overcome challenges [12, 13]. Unfortunately, these workarounds may impact on the patient and the system [14]. Therefore, understanding how healthcare workers respond to these challenges is essential.

A workaround is an intentional adaptation, improvisation or change to an existing work system in order to overcome, or lessen the impact of obstacles, exceptions, anomalies, mishaps, established practices, management expectations, or structural constraints that are perceived as preventing that work system or its actors from achieving a desired organizational or personal goal [15] (p1044). Workarounds temporarily help to overcome a problem by using non-standard/ informal means [1, 15] whereby individuals use an alternative work process instead of the existing process [16]. As a result, the goal is met but the issue causing the problem is not resolved or eliminated [15]. Preconditions for the existence of a workaround are the presence of a process/standard in the system; the presence of challenges with the standard, with actors, still expected or assigned to achieve a goal; and being motivated to continue performing despite the obstacle. Thus, there is evidence that workarounds are performed for a functional purpose and not necessarily as a form of oppositional behaviour [17]. Therefore,instead of disciplining individuals who work around the system, actors should be involved in analysing, changing, and improving the system [18],because there are factors in the environment contributing to this workaround.

Previous studies have identified workarounds in medication administration processes [19]. For example, in a study to examine workarounds associated with the implementation of an electronic medication administration record of nursing homes in Columbia [13], nurses employed workarounds due to the introduction of new and unfamiliar technology. The nurses perceived the use of this technology as a block to their work because they found the new system to be inefficient. Other obstacles contributing to workarounds are blocks in workflow, work demands, poorly designed work systems, organisational policies, protocols and people [14, 19, 20].

While workarounds may have a potential efficiency benefit through a reduction in the number and complexity of tasks [16], unintended, negative consequences of workarounds may also occur [20]. Workarounds create hazards, inefficiencies or errors and impact subsequent steps or activities, and cause noncompliance with management intentions [15]. Efficiency is reduced when a workaround increases the number and complexity of tasks making the workaround more labour intensive than the original work process [16]. Introducing shortcuts by removing a few steps or activities may lead to the loss of an essential phase in the process, potentially putting the patient at further risk [16]. Literature recognises workarounds, but there is a dearth of studies of workarounds and their causes [15, 21]. Some studies have explored nurses’ workaround but limited their focus to the use of technology in medication administration processes [2, 14, 16, 22, 23]. Other studies have explored physician workarounds in information systems [24] but with little understanding of their nature, extent, or outcomes [19]. For example, Koppel et al. [23] identified workarounds related to the use of Bar Code Medication administration technology such as giving partial doses but documenting full doses and documenting medication before administration.

Although previous studies have made efforts to understand workarounds, they are mostly from developed countries where the use of technology is being adopted. Debono et al. [14] found that most studies about workarounds were conducted in the US, underscoring the need for workaround studies in lower- and middle-income countries. Malawi is a resource-poor setting where medical technology use in medication administration is limited or underutilized, and such a workaround study has not been done. Additionally, the present study focused on antibiotic stewardship where there is a dearth of such studies and extends the knowledge of workarounds to resource-poor countries, where workarounds are likely due to factors such as material and human resource challenges. The present study examines workaround behaviors that nurses and doctors employ to address the challenges encountered during their antibiotic stewardship efforts at a tertiary hospital in Malawi. It also examines the impact of such behaviors.

## Methods

### Study context

We conducted this study in male and female medical wards of a tertiary hospital. Both wards have a bed capacity of 67 patients but the numbers of patients requiring treatment exceed this capacity frequently. Patients with acute conditions such as pneumonia, meningitis and sepsis populate these wards, and the majority of patients receive antibiotic therapy.

### Design

This study is part of a larger mixed methods case study conducted to understand nurses’ role in antibiotic stewardship and the challenges they face with the aim of developing context-specific antimicrobial stewardship nursing interventions. In this paper, we report a qualitative case study [25] to understand the phenomenon of workarounds during the patient antibiotic management process. We used multiple strategies and triangulated different data sources to illustrate how different types of workarounds occur; contributing factors; and the perceived impacts of workarounds on patient care.

### Participants and data collection

We purposively sampled participants for the focus group discussions. We held the first focus group discussion with pharmacists and laboratory technologists (*n* = 8); the second group comprised senior medical doctors (*n* = 6), and the third focus group included junior doctors (n = 6). The purpose of the focus groups was to learn about their experiences with nurses working in adult medical wards caring for patients on antibiotic treatment. A focus group guide was used to facilitate the discussion (Additional file 1).

We also conducted participant observations of nurses’ antibiotic stewardship practices in the two medical wards using an observation guide (Additional file 2). The primary author collected data while participating in some aspects of care. Participant observations focused on four events that were purposively selected based on common antibiotic management encounters: nurses’ shift change handover reports (*n* = 10), antibiotic preparation (*n* = 13), antibiotic administration (*n* = 49 cases) and ward rounds (*n* = 7). We conducted participant observations on selected days during day and night and weekend shifts. The purpose of the observations was to understand nurses’ practices and responses to challenges when managing patients on antibiotics. Barriers identified during focus group discussions and participant observations were followed up with interviews with purposively selected nurses (*n* = 13) using an interview guide (Additional file 3). The interviews helped to document the specific nature, contributing factors and perceived impacts of workarounds on patients and the health care system.

### Analysis and rigor

Inductive and deductive approaches to thematic analysis were used [26]. Focus group transcripts and observation field notes (transcripts) were first read and re-read to get a sense of the meaning as they related to antibiotic stewardship practices, challenges/obstacles, and how nurses and doctors overcame them. Data was transported into Nvivo 10 for better management during analysis. Transcripts were coded first using an inductive approach where we openly coded each relevant theme. We organized the data and identified themes regarding the nature of the blocks, how participants performed workaround, and the participants’ opinion of the impact of workaround using the concepts ‘challenges/barriers’ and ‘workaround’.

To establish rigour, we used member checking and data source triangulation [27]. For member checking, CM discussed our data interpretation with the nurse participants who confirmed the findings. We triangulated doctors’ and nurses’ perspectives of workarounds and later confirmed and clarified the data in the follow-up interviews with the nurses.

## Results

Workaround findings emanated from focus group discussions, observation of nurses during the two events namely: shift change handover reports and antibiotic preparation; and nurse interviews. The demographic data of participants is presented in Additional file 4. Through an iterative analysis process, we observed six antibiotic management workarounds that we organised in two broad categories: 1) Taking shortcuts by altering a procedure [15] and 2) Using unauthorised process steps or adding new steps to the procedure [23].

### Category 1: Taking shortcuts by altering a procedure

Three antibiotic stewardship workarounds were identified:

- Nurses using saline water from a 500 ml bottle instead of the pre-packaged water for injection to reconstitute antibiotics.

During antibiotic preparation (reconstitution) nurses chose to alter a procedure by finding an alternative to the diluents available because the standard diluents took too long to draw into a syringe.

*84 vials of 500 mg cefotaxime each are placed on the table with a 500mls normal saline bottle as diluents and boxes of 10mls syringes.*

The researcher asked why you, the nurse, used the water from the 500 ml bottle as diluents instead of the pre-packaged water provided?

*The nurse says the diluents from the small vial is difficult and takes too long to draw (male ward)*

During antibiotic preparation, antibiotics are supplied with pre-packaged diluents enough for each vial of the antibiotic. From the nurses’ experience, this standard seems to impose a workflow block/delay as it takes a long time to draw water from this vial unlike using a 500 ml saline bottle as a diluent because it is faster to draw from this bottle.Normal saline is used for intravenous therapy and can only be substituted as a diluent if the pre-packaged diluent is not available.

b) Preparing and drawing a larger quantity of intravenous antibiotics to save time

During antibiotic reconstitution, nurses prepared and drew more antibiotics for the next two medication rounds.

*One hundred and sixteen (116) vials of Cefotaxime were prepared and drawn. The male nurse puts some drawn Cefotaxime in an improvised paper tray and stores it in the fridge. (Male ward)*

*Cefotaxime is placed on the table along with syringes and water for injection. Two hundred (200) vials of 500 mg are diluted with 2mls of water. (Female ward)*

The antibiotics prepared each time were more than required for that administration schedule.

During the follow-up interview participants stated the following reasons for such practice:

*We still do (preparing more antibiotics at once) because we keep them in the fridge, but it’s not recommended. We try to relieve ourselves because of workload so we end up doing shortcuts. (Male nurse 12, female ward)*

Nurses seem to be aware of the standard protocol of reconstituting antibiotics during administration time, but encounter challenges due to staff shortages and time constraints.

c) Giving suboptimal antibiotic medication teaching to patients for ease of understanding

We found that nurses chose to alter a procedure by making shortcuts during the teaching of patients about oral antibiotics:

*The common mistake that we make is that aaa we tell them (patients and relatives) to give antibiotic in the morning, afternoon and evening because we just assume in the morning they give at six, in the afternoon they can give at two, and in the evening, they can give at six. (Female nurse 05, male ward)*

*We just say in the morning, in the afternoon and in the evening. (Male nurse 12, female ward)*

In trying to explain why they teach this way, one participant said:

*And that might be because of our situation here. Level of understanding is different; mostly our patients are those who can’t read and write so if you tell them two pm they might not be able to know that this is two pm; I should take the drugs unless if we are giving the drugs ourselves.(Male nurse 12, female ward).*

The teaching only focused on the frequency and not the specific time intervals which might lead to a patient taking the antibiotic at the incorrect times. Patients’ level of understanding was reported as a block contributing to the workaround.

### Category 2: Using unauthorised process steps

Three workaround practices were identified under this category:

- Nurses using patients’ relatives as an aid to facilitate parenteral antibiotic administration

This workaround involved altering the best practice of medication administration, where the nurse collects and administers the parenteral medication at the bedside. Nurses perceive this practice as time-consuming because of the high patient to nurse ratio (typically 60:2 nurses) and the limited space for nurses to move bed by bed when wards exceed their bed state capacity. As a result, nurses allowed the patient’s relative to keep the syringe with the medicine in advance of administration by the nurse:

*We tell the guardians (patients’ relatives) so the syringe will be put in a cup that is clean they are told not to put the drug anywhere. It’s just that we are understaffed. (Female nurse 5, male ward)*

Related to this is the practice of one nurse signing (documenting) for the medication before it is administered and another nurse administering, thus challenging the principle of accountability.

*Maybe time management, because you take every injection you give, you come, you go, you come. It’s quite costing in terms of energy and you end up being tired. That’s why we always divide ourselves you be on the drug trolley to document and you will be moving around to inject…(laughter). (Male nurse 01, male ward)*

*During preparation of the drug, we may prepare the drug but it may stay longer. For example, we may take the drug to the bedside we find that it has been like a tradition that we take the drug, we put it on the bedside and we leave it for someone to give the drug. (Male nurse 11, female ward)*

Nurses reported that they gave the antibiotic to patients’ relatives in advance, and then the nurses follow later to administer the medication. Another workaround included one nurse signing and another administering the medication. The reason for this shortcut is to save time and energy by preventing frequent trips between patients and the drug trolley.

b. Equalisation of care: Administering a low dose or less frequent dosing schedule***.***

Nurses administered a low dose of antibiotics different from the one prescribed and documented it on the original prescription dose different from the one given:

*We will be giving 1 g instead of 2 g because the antibiotic will not be enough for all the patients. (Nurse, male ward)*

*Even those prescribed 2 g we gave 1 g yesterday so that it’s enough for all patients. (Nurse, female ward)*

In instances where the antibiotics are insufficient for all the patients, nurses administered a lower dosage to ensure all patients received antibiotics. A second factor driving this practice was that when doctors prescribed different schedules (higher doses) for the same diagnosis and similar presentations; nurses adjusted the higher doses for the benefit of all patients. Thus, if the antibiotic supply is low (block one) and patients antibiotic schedules for the same diagnosis and symptom presentation different (block 2), nurses adjusted the prescription on their own to match other patients’ treatment plans.

c. Doctors prescribing an antibiotic to best suit nurses’ situations

Nurses’ challenges with adhering to more frequent dosing schedules (three times and four times a day), were observed to have influenced doctors’ prescription behaviours. During focus group discussions, pharmacists questioned why doctors appear to mostly prescribe Ceftriaxone when the pharmacy has other antibiotics such as crystalline penicillin and chloramphenicol. During the doctors’ focus group discussion, participants mentioned that they use Ceftriaxone because it is given less frequently and it is easier for the nurses to administer:

*I have very often the feeling that I prefer grabbing the drug even if it’s not the best choice if it’s just once daily because as soon as you start prescribing the four times daily it’s more probable that they don’t receive anything. (Medical doctor14).Others echoed yea*

*So I will give you an example: We prefer doxycycline instead of erythromycin because if with erythromycin you will get it four times daily you will be lucky for a patient to get more than three doses with erythromycin so anything that requires a typical cover you end up using doxycline because it’s twice daily. When the guidelines tell us that we should use benzyl penicillin plus chloramphenicol for severe pneumonia four times daily for each of those drugs aaa they would end up maybe getting two doses so we tend to err on the side of ceftriaxone.(Medical doctor 14)*

*When you give some antibiotics very closely it’s difficult for the nurses. For example, Ceftriaxone; we love Ceftriaxone because it is only once or twice a day and it cannot be missed when the ward is busy and is understaffed. (Junior doctor 31)*

Doctors are concerned that when they order antibiotics with short interval dosing schedules, it is difficult for nurses to adhere to this prescription schedule. The doctors work around this obstacle by prescribing the less frequently-given antibiotics to suit the nurses’ situation, thereby making sure patients receive medication according to prescriptions.

### Impact of the workarounds: Patient safety concerns

Although workaround behaviours are widely used and may be beneficial, nurses acknowledged their impact on the safety of their patients. Nurses also noted that workarounds violate some of the ‘Five Rights’ to medication administration such as the right drug, right dose and right time.

#### The impact of preparing and drawing parenteral medications in advance

*The one giving injectables is not the one who is drawing the drug. You can easily mix the drugs even though we say we are used to that this is Ceftriaxone this is promethazine but you can easily mix them. (Female nurse10, female ward)*

*Sometimes you find that you have prepared 2 g and there is another patient who is supposed to receive 1 g you never know the nurse who is there may give 2 g instead of 1 g because the drug is already in the syringe. (Female nurse 04,male ward)*

#### The impact of involving patients’ relatives to handle antibiotics

*Some patients come without a guardian and for someone who is really sick may not be able to come and receive the drugs and this is really a big problem. Sometimes they cannot even shout to the nurse to say you haven’t given me the drug so it’s really a problem you wouldn’t blame anyone. It’s just that we are understaffed. (Female nurse 05, male ward)*

*Mm the challenge is that some of the medications are forgotten because you give the guardian the medication and you forgot that this patient has been given the intravenous antibiotic and then you give the medication when the time has gone. (Female nurse 09, female ward)*

It seems nurses are aware of the negative impact or errors these workarounds may have on patients’ safety. In the first workaround, the patient may get the wrong medication or the wrong dose because a different nurse prepared the antibiotic while the nurse giving it may not know how it was prepared. Similarly, the practice of relying on patients’ relatives during antibiotic administration may cause errors such as missing a dose or receiving the medicine at the wrong time.

Based on the identified challenges and the workarounds; study participants were asked to suggest interventions to help improve the practices. The following themes/topics were the most commonly suggested as interventions for improvement: antibiotic administration guidelines/ protocols; in-service training; supervision, auditing/ monitoring and evaluation of practices.

## Discussion

The challenges related to caring for patients on antibiotics in this setting have exposed workarounds. Nurses and doctors are communicating unwritten, but widely accepted, cultural practices such as making shortcuts, improvising, and deviating from standards evident in the activities of prescription, preparation, storage, administration and patient antibiotic teaching.

### Making short cuts by altering a procedure

Altering a procedure is performing activities in a different way such as skipping steps, adding steps, changing the sequence of steps or using different techniques or resources because of the perceived burdensome nature of prescribed processes and activities [15]. These prescribed processes, while theoretically and ethically sound, may be perceived as unrealistic and burdensome because they are difficult to achieve/maintain in practice. Firstly, concerning altering work process resources, nurses in the present study substituted the first resource (pre-packaged diluents) with another but equally recommended resource (saline water) during antibiotic reconstitution because the formal procedure prolongs the process of drawing the diluents, demanding more time in an already under-staffed environment. Such a practice could be related to a type of workaround “*Design and implement new resources”* [15], a strategy to ensure the timely reconstitution and administration of antibiotics. This practice may also be related to nurses’ skill gap in drawing diluent from the small vial. Indeed, obstacles to doing work in a preferred manner may come from the knowledge and skills of actors, available information, and the features and capabilities of technology [15]. Despite that the saline diluent is equally recommended, the practice remains a deviation from the standard for appropriate use of resources. Secondly, for the perceived burden of the prescribed work process, nurses in the present study worked around the obstacle of time constraint by using the “quick fix” of preparing and drawing more parenteral antibiotics at once to save time later [15]. This workaround is comparable to Morath’s [28] report of a nurse caring for five patients, all requiring administration of medications twice during the shift. The nurse adopted a workaround by withdrawing each patient’s medications for medication administration rounds at one time. The nurse would then store the medicines, for example in the uniform pocket, until needed and could efficiently administer the specified medications to each patient without returning to the medication trolley a second time. However, this quick fix can have disastrous consequences. For example, Pape [29] reported a case where a nurse caring for a patient in an acute ward during a night shift had a heavy workload and adopted a workaround by preparing and placing two different medicines, insulin and an antibiotic, next to each other in advance, and mistakenly gave insulin instead of the antibiotic. In this study, there could be a risk of mixing up different antibiotics or other medicines. Both instances deviate from standard procedure, which is for the nurse to prepare the antibiotic during the scheduled time and administer patient by patient.

Lastly, workarounds in this category are related to patient challenges with an understanding of medication times. Nurses take shortcuts during patient teaching by using common terms such as morning, afternoon and evening to denote frequency without talking about dose interval periods. This workaround uses different information (to the one recommended) because of problems with quality, timeliness, completeness, or cost of the officially recommended information [15]. Though the information given to patients using a shortcut is not wrong, it is inadequate. For example, the nurses attempt to work around patient ignorance by omitting information about medication time intervals. We must also consider the possibility of nurses’ ignorance of the importance of adhering to time intervals to minimize antibiotic resistance. It seems that both patient and nurse competency factors have contributed to this workaround. Therefore, work processes, patient-related factors and professional factors contribute to workarounds [2, 14]. There is a need to provide stricter instructions to patients about the time intervals between antibiotic doses to optimize the antibiotic efficacy and prevent resistance.

### Using unauthorized process steps

The first workaround under this category is nurses involving patients’ relatives to facilitate antibiotic administration. This practice is similar to Alter’s [15] description of different unqualified participants being allowed to do the work because the people who should do the work are unavailable. A multidisciplinary study to understand workaround occurrences during the use of Bar Code Medication administration in five large hospitals (with a total bed count of 1400) in the US found that due to the challenges with technology, nurses and clinicians worked around this problem by omitting some steps in the process, performing some steps out of sequence and using unauthorized steps [23]. Though the use of unqualified staff was not the issue in Koppel’s [23] study, the two findings demonstrate the different ways that nurses can use unauthorized process steps to workaround medication administration challenges.

The second workaround was nurses equalizing care by administering a low dose or less frequent dosing schedule without consulting the prescribing doctor. It seems that a short supply of antibiotics influence the nurses’ action of adjusting the dose. The Bar Code Medication Administration study also reported the workaround of giving a different dose from the one prescribed [23], showing that where a half medication dose was available, the nurse scanned the medication twice to indicate administration of the full dose without consulting the prescriber. Our study, as with Bar Code Administration found that poor communication between members of the health care team contributes to this workaround. Similarly, Jordan et al. [30] in Canada found that poor communication with physicians contributed to nurses' workarounds with nurses choosing not to continue with physicians’ orders. In Jordan’s study the nurse viewed herself as capable of recognizing an unsafe order but the dismissive manner in which the physician disagreed with the nurse, created a workaround situation. When nurses approached doctors for clarification, doctors responded by saying “just do it” or “just push it” (p 69) leaving nurses to create workarounds to address issues. Although poor communication is a feature of our study, nurses in our study, unlike Jordan’s research, did not seek clarification from the doctor about the prescription in question. A study examining a standard work process in intensive care units of four hospitals found three main barriers: information exchange, information entry, and internal supply chain [2]. Nurses reported working around the block in those situations by making decisions without the physician’s input.

Lastly, analysis of nurses’ practices showed that the nurses’ behavior influenced doctors’ workaround behaviors. Doctors reported that they prefer prescribing an antibiotic with a less frequent dosing interval because of nurses’ poor compliance to more frequently dosed antibiotics. Clinicians implement workarounds as a way of responding to the complexity of care within a system [14]. Thus, environmental and task-related factors [23] have contributed to this workaround. Furthermore, communications may have a role in the use of workarounds [31]. The workaround of prescribing a less frequent dosing schedule, such as once or twice a day, seems to benefit not only the patients who receive the intended care but also the nurses whose workload is made easier. Despite this workaround being a solution from the doctors’ perspective, it bypasses the safety features built into prescription guidelines. In this case, doctors bypassed the safety feature of following the recommended prescription guideline by using the less common/recommended antibiotic. This workaround is exemplified in the present study as doctors avoid confronting nurses about their poor adherence to the more frequent dosing schedules because doctors seem to understand the nurses’ challenges. We may also speculate that doctors possibly want to maintain a professional relationship with the nurses. This type of workaround is related to what Alter [15] calls a plot for mutual benefit.

### Impact of workarounds from the nurses’ perspective

Workarounds may be expressed as unusual, important or, sometimes as questionable, undesirable, hazardous, or even unethical behaviors [15]. They may have an impact on patients’ safety, improvement efforts and cost as has been noted in the present study. Direct effects of workarounds include the continuation of work, despite obstacles and compliance with management intentions [24]. Although evidence suggests some positive impacts of workarounds, nurse participants in the present study perceived only negative impacts on the ‘Five Rights’ to medication administration [32].

Similarly, Koppel et al. [23] stressed that workarounds might lead to giving the wrong medication to the wrong patient or giving the wrong dose and route. Also, the practice of allowing patients’ relatives to handle/keep antibiotics in the syringe potentially risked infection as the patients’ relatives might not handle the syringe properly. All this has the potential to contribute towards antibiotic resistance, toxicity and infections and may lead to a prolonged hospital stay.

Furthermore, workarounds may hinder quality improvement efforts as attempts to alter the system to deal with the root causes may not occur [1]. For example, doctors initiate workarounds as a way of problem-solving to accommodate nurses, and care continues. This practice may not help nurses to improve adherence to the more frequently given antibiotics. Similarly, some nurses may not develop their skill in drawing diluents from the pre-packaged vials as they will continuously rely on using the alternative saline bottle.

Workarounds conceal the magnitude of nursing and antibiotic supply shortages because nurses continuously meet their goals of fulfilling the task at hand as they give a less dose (which also illustrates a task-oriented attitude). This improvisation or finding an alternative as a solution to a problem may have an immediate benefit, but causes the magnitude of the problem to go unnoticed and little is done to remove the system barrier [31].

Lastly, workarounds may have cost implications. The practice of prescribing the less frequently administered antibiotic may lead to underutilization of other antibiotics available in the pharmacy. The same may be the case with the use of the 500 ml or one-litre saline as diluents. Once the saline bottle in opened, there is more left, and it may not be safe to be used later due to infection risks. As a result, it is usually wastefully discarded.

### Strengths and limitations

The researchers acknowledge several limitations to the study. Being a case study based in two wards in one hospital limits the generalization of findings across diverse settings. However, the description of the study context supports the transferability of these findings in similar environments. Given its exploratory nature, the study presents the first step in understanding the phenomena of workaround within the Malawian context. Triangulation of data sources from doctors, pharmacists, and nurses, and the use of multiple methods namely focus group discussion, participant observation and interviews, increases confidence in the analytic process and study findings.

Studying workarounds by observing or interviewing can lead to an underestimation of workarounds because of the Hawthorne effect [33], where participants may change behavior to suit the observer or interviewer situation [27]. The findings, therefore, may be limited as there could be more to these behaviors. We only documented workarounds that were reported, observed and confirmed by front-line workers.

In the present study, the researchers did not examine the participants’ understanding of internal factors driving workarounds, or the processes of the institutionalization of workarounds in their daily practice, or the managers’ awareness and perception of workarounds. Further research on the contextual attributes of workarounds [34] will illuminate whether they are avoidable or unavoidable, essential, temporary or reutilized, deliberate or unplanned [35].

## Conclusion

The study has provided new insight into critical practical examples of how nurses work around workflow blocks encountered during patient antibiotic management. We identified two categories of workaround: Taking shortcuts where nurses substituted original diluents with saline, prepared larger quantities of antibiotics in advance and taught patients in shortcuts. The second category is where unauthorised process steps were used such as involving patient relative in the antibiotic administration process, giving a lesser dose than the one prescribed and doctors prescribing an antibiotic best suiting the nurse’s work-load. Nurses identified negative impacts of some of these behaviours. In the context of broader issues, workarounds are not the nurses’ or doctors’ fault; they use workarounds to do the best they can in the situation. For example, medicine shortages may not be a problem in a developed country, but in our context, this is a genuine and massive system issue facing nurses, leading to workarounds. Besides, understanding these workarounds as a means of problem-solving is essential to understanding their implications for patient antibiotic management safety. Eliminating or reducing the blocks in the system would minimise workarounds. There is a need to improve coordination and communication among nurses, pharmacists, and physicians (multidisciplinary teamwork). Training healthcare workers about workarounds and how potentially dangerous and unethical these workarounds can be is also important. Nurses should be empowered to advocate for patients and avoid, where possible, using ad hoc solutions to address the challenges. Management should also be aware that workarounds in these contexts are better than nothing and not outright bad but should consider the complex ethical issues that come with these workarounds.

## Additional files

Additional file 1: Focus group discussion guide. A data collection tool used during focus group discussion with pharmacists and laboratory technologists (*n* = 8), senior medical consultants (*n* = 6); and junior doctors (*n* = 6). The purpose of the focus groups was to learn about their attitudes and experiences with nurses working in adult medical wards in relation to caring for patients on antibiotic treatment. (DOCX 17 kb)

Additional file 2: Observation guide. A data collection tool used during participant observations of nurses’ antibiotic stewardship practices in the two medical wards. Observations focused on four events: nurses’ shift change handover reports (*n* = 10), antibiotic preparation (*n* = 13), antibiotic administration (*n* = 49 cases) and ward rounds (*n* = 7). The purpose of the observations was to understand nurses’ practices and responses to challenges when managing patients on antibiotics. (DOCX 16 kb)

Additional file 3: Interview guide. A data collection tool used during follow up interview with some observed nurses (*n* = 13). The purpose of the interview was to document the specific nature, contributing factors and perceived impacts of workarounds on patients and the health care system. (DOCX 14 kb)

Additional file 4: Demographic data of participants. Demographic data of participants in focus group discussion, observation and follow up interviews. (DOCX 19 kb)
