# Supplementary material for: An exploration of workarounds and their perceived impact on antibiotic stewardship in the adult medical wards of a referral hospital in Malawi: a qualitative study
Source: BMC Health Serv Res. 2019 Jan 23;19:64. doi: 10.1186/s12913-019-3900-0 (PMC6345002; doi:10.1186/s12913-019-3900-0)
Supplement: Supplementary file 1 — Focus group discussion guide. A data collection tool used during focus group discussion with pharmacists and laboratory technologists (n = 8), senior medical consultants (n = 6); and junior doctors (n = 6). The purpose of the focus groups was to learn about their attitudes and experiences with nurses working in adult medical wards in relation to caring for patients on antibiotic treatment. (DOCX 17 kb) [file 12913_2019_3900_MOESM1_ESM.docx]

# Additional file 1: Focus group guide

I.D Number.....…................... Date of FGD.............................Place of FGD..........................

Number of Participants: Drs….. / Pharmacists……/Microbiologists ......../Total.................

Name of the interviewer/ moderator....................................................

Name of the recorder / note taker…………………………………..

**Introduction:**

The interviewer will introduce and explain the purpose of the discussion. Explanation will be that the discussion focuses on your perceptions and experiences of nurses’ role in antibiotic stewardship and the challenges.

**Perception towards Antimicrobial resistance**

What are your thoughts about antimicrobial resistance?

How big is the problem of antimicrobial resistance? How often do you encounter in patients with problems of antimicrobial resistance in your department?

What can go wrong for antimicrobial resistance to occur?

**Perception of Antibiotic Stewardship**

What do you understand about antibiotic stewardship? (Will define if unclear)

Who do you think should be involved in Antibiotic stewardship? Why?

What are the key components needed to develop AMS interventions e.g. guidelines, protocols education to nursing staff and families,

**Perception/experience with nurse involvement in Antibiotic Stewardship Interventions.**

I understand there is no established Antimicrobial stewardship program at this hospital but you and nurses are involved in areas of antimicrobial management activities that are related to Antimicrobial stewardship. The current project is about developing a nurse focused Antibiotic stewardship intervention/s:

- Do you have any processes or protocols/guidelines to help in proper management of antibiotics/patients on antibiotics? Does it reflect nurse involvement?
- Do you think a nurse has a role to play in this? Why? Or how do you see the role of nurses in facilitating the Antibiotic Stewardship Program?
- How/Where can a nurse participate in Antibiotic stewardship? (Probe: Do nurses have a role in antibiotic management during patient admission; ward rounds, during specimen processing, during discharge?)
- What are the areas that nurses do well in addressing appropriate use of antibiotics? Where are they not doing well?
- What do you think makes them not function well in other areas?
- How do you think the doctors/pharmacists/laboratory technologists in general would support Antibiotic stewardship program that has a nursing focus for adult in patients?
- What would you imagine might be the major barriers in implementing antibiotic stewardship with a nurse focus? What solutions do you suggest? (Explore the facilitator/enables)

**Any other issues**

Please explain any concerns or ideas you have about nurse involvement in antibiotic management.

**For Doctors only**

**Organizational culture**

- How do you feel about nurses’ patient assessments (prompt issues about nurses’ knowledge in recognizing infections/timeliness in reporting)?
- How do the opinions/attitudes of nursing staff influence your antibiotic prescribing?
- Who follows up pathology/microbiology results (sent directly to you, rely on nurses)?

**Any other issues**

Are there any other issues that you feel we haven’t talked about that you would like to mention?
