# Supplementary material for: An exploration of workarounds and their perceived impact on antibiotic stewardship in the adult medical wards of a referral hospital in Malawi: a qualitative study
Source: BMC Health Serv Res. 2019 Jan 23;19:64. doi: 10.1186/s12913-019-3900-0 (PMC6345002; doi:10.1186/s12913-019-3900-0)
Supplement: Supplementary file 2 — Observation guide. A data collection tool used during participant observations of nurses’ antibiotic stewardship practices in the two medical wards. Observations focused on four events: nurses’ shift change handover reports (n = 10), antibiotic preparation (n = 13), antibiotic administration (n = 49 cases) and ward rounds (n = 7). The purpose of the observations was to understand nurses’ practices and responses to challenges when managing patients on antibiotics. (DOCX 16 kb) [file 12913_2019_3900_MOESM2_ESM.docx]

# Additional file 2: Observation Guide

**Context of the observation: Antibiotic management during these events:**

**(Indicate name of event, Date, Ward setting, start and finish time.)**

| **Events:**   - Nursing Handover report - Antibiotic preparation - Antibiotic administration - Ward round |
| --- |

**What to observe: Expected tasks, communications.**

Instructions to Observer: Write down in your note book as field noted actions you see and comments you hear under the following headings:

| - Antibiotic management discussions, interactions you hear during the events i.e. antibiotic management ward round interaction between nurse and doctor. - Observe the activities how they are done i.e. Storage and preparation of the antibiotics. - Note presence of other elements in the environment that may be impacting on for example, antibiotic preparation (such as number of antibiotics to be prepared), any other task the nurse is undertaking at the same time for example antibiotic preparation activity is occurring, attending to the request of another patient, responding to another health professional etc. - Nurses activities, interactions or what they say in relation to antibiotic management. Any reflection of decision making, clinical judgment, caring practice and advocacy role during the observed event. - Observing what does not happen – Any missed activity, explore why. - Length of time of observation of each event. |
| --- |
