# Supplementary material for: An exploration of workarounds and their perceived impact on antibiotic stewardship in the adult medical wards of a referral hospital in Malawi: a qualitative study
Source: BMC Health Serv Res. 2019 Jan 23;19:64. doi: 10.1186/s12913-019-3900-0 (PMC6345002; doi:10.1186/s12913-019-3900-0)
Supplement: Supplementary file 3 — Interview guide. A data collection tool used during follow up interview with some observed nurses (n = 13). The purpose of the interview was to document the specific nature, contributing factors and perceived impacts of workarounds on patients and the health care system. (DOCX 14 kb) [file 12913_2019_3900_MOESM3_ESM.docx]

**Additional file 3: Interview guide.**

**Nurse’s perceptions and experience with antibiotic stewardship practices undertaken during observation.**

- How would you define antibiotic stewardship in relation to antibiotic management activities you undertook?
- Can you please comment on the extent to which you think that you demonstrated antibiotic stewardship practices in relation to medication activities you undertook during handover/antibiotic preparation/administration/ward round?
- Was there anything that you think was not consistent with your beliefs/perception about antibiotic stewardship during preparation/administration?
- What factors influenced your antibiotic management during the observation period (specify the event)?
- What challenges do you experience in adhering to antibiotic regimen for inpatients in your ward?
- What aspects of current antibiotic management system in general which you think are a challenge and may need further improvement or solution?
