# Supplementary material for: An exploration of workarounds and their perceived impact on antibiotic stewardship in the adult medical wards of a referral hospital in Malawi: a qualitative study
Source: BMC Health Serv Res. 2019 Jan 23;19:64. doi: 10.1186/s12913-019-3900-0 (PMC6345002; doi:10.1186/s12913-019-3900-0)
Supplement: Supplementary file 4 — Demographic data of participants. Demographic data of participants in focus group discussion, observation and follow up interviews. (DOCX 19 kb) [file 12913_2019_3900_MOESM4_ESM.docx]

# Additional file 4: Demographic characteristics of health worker participants

**Demographic data of Focus group participants**

| **Pharmacists and laboratory technologists** | | | | |  |
| --- | --- | --- | --- | --- | --- |
| **ID number** | **Sex** | **Age Range** | **Qualification** | **Work experience** |  |
| 1 | M | 23 – 51 years | Diploma in laboratory technician | 14 years |  |
| 2 | M |  | BSc in medical laboratory technology | 5 years |  |
| 3 | F |  | BSc in medical laboratory technology | 2 years |  |
| 4 | F |  | Bachelor of pharmacy | 10 months |  |
| 5 | M |  | Bachelor of pharmacy | 10 months |  |
| 6 | M |  | Diploma lab technician | 1 year 1 month |  |
| 7 | M |  | Bachelor of pharmacy | 10 months |  |
| 8 | M |  | Bachelor of pharmacy | 10 months |  |
| **Medical Doctors (Registrar and Medical Consultants)** | | | | |  |
| 09 | | F | 32 – 58 years | Medical Doctor | 8 years |
| 13 | | F |  | Medical Doctor | 1 year (in Malawi) |
| 10 | | F |  | Medical Doctor | 4 years |
| 15 | | M |  | Medical Doctor | 35 years |
| 12 | | M |  | Medical Doctor | 4 years |
| 14 | | F |  | Medical Doctor | 21 years |
| **Medical Interns** | | | | | |
| **ID Number** | | **Sex** | **Age Range** | **Work experience** |  |
| 34 | | F | 24 – 26 years | 16 months |  |
| 40 | | F |  | 16 months |  |
| 30 | | M |  | 16 months |  |
| 36 | | F |  | 16 months |  |
| 35 | | F |  | 16 months |  |

**Demographic data of medical ward nurses Observed.**

| **Ward** | **Number of nurses** | **Age Range** | **Qualification** | **Gender** | **Work experience** |
| --- | --- | --- | --- | --- | --- |
| Male medical ward | 11 | 25-38 years | 4 Diploma  3 Degree  4 Certificate (technician) | 7 female  5 male | Range 1-44 years |
| Female medical ward | 12 | 24-56 years | 2 Diploma  1 Degree  9 Certificate and technician | 8 female  4 male | Range 1-13 years |

**Demographic data of nurse participants interviewed**

| **ID number** | **Age Range** | **Sex** | **Nursing Qualification** | **Work experience** |
| --- | --- | --- | --- | --- |
| Male Medical Ward | | | | |
| 01 | 25 – 38 years | M | Diploma | 5 months to 13 years |
| 02 |  | F | Degree |  |
| 03 |  | F | Degree |  |
| 04 |  | F | Diploma |  |
| 05 |  | F | Degree |  |
| Female Medical Ward | | | | |
| 06 | 24 – 68 years | F | Degree | 1 to 44 years |
| 07 |  | F | Diploma |  |
| 08 |  | M | Certificate (Technician) |  |
| 09 |  | F | Diploma |  |
| 010 |  | F | Certificate (Technician) |  |
| 011 |  | M | Certificate (Technician) |  |
| 012 |  | M | Certificate (Technician) |  |
| 013 |  | F | Certificate (enrolled NM) |  |
